# Soft Tissue Facial Morphology in Growing Patients with Different Occlusal Classes

**DOI:** 10.3390/jpm14101042

**Published:** 2024-10-07

**Authors:** Niccolò Cenzato, Marco Farronato, Francesco Carlo Tartaglia, Lucia Giannini, Angelo Michele Inchingolo, Gianna Dipalma, Cinzia Maspero, Francesco Inchingolo

**Affiliations:** 1 Department of Biomedical, Surgical and Dental Sciences, University of Milan, 20122 Milano, Italyluciamariachiaragiannini@gmail.com (L.G.); 2 Fondazione IRCCS Ca’ Granda Ospedale Maggiore Policlinico, 20122 Milan, Italy; 3 Department of Biomedical Sciences, Humanitas University, Via Rita Levi Montalcini 4, 20072 Pieve Emanuele, Italy; francesco.tartaglia@st.hunimed.eu; 4 Department of Interdisciplinary Medicine, University of Bari “Aldo Moro”, 70124 Bari, Italy; gianna.dipalma@uniba.it (G.D.); francesco.inchingolo@uniba.it (F.I.)

**Keywords:** facial morphology, skeletal classes, facial scan, morphometry

## Abstract

Introduction: The study of facial profiles in the dental field is very important for the diagnosis and the dental and orthodontic treatment plan. The aim of this study is to analyze the three-dimensional morphology of the faces of 269 growing patients with Class I and II occlusions, focusing on children aged between 6 and 9 years old. The analysis was conducted using a non-invasive computerized system, which allowed for the automatic collection of facial landmarks and the subsequent reconstruction of three-dimensional coordinates. Materials and methods: The sample comprised 269 children within the specified age range. Each child’s facial features were captured using the non-invasive computerized system, which utilized two infrared CCD cameras, real-time hardware for label recognition, and software for three-dimensional landmark reconstruction. Sixteen cutaneous facial landmarks were automatically collected for each participant. From these landmarks, 10 angular and 15 linear measurements, as well as five direct distance rates, were derived. The mean values for each age class were calculated separately for children with bilateral Angle Class I occlusion and compared with those for children with bilateral Class II occlusion. In all children, the left and right occlusal classes were measured as suggested by Katz. Results: The analysis revealed notable differences, primarily in the three-dimensional angular measurements between children with Class I and II occlusions. Specifically, Class II children exhibited more convex faces in the sagittal plane and a less prominent lower jaw compared to Class I children. However, no significant differences were observed in linear measurements, except for the lower facial height rate, which varied inconsistently across age groups between the two occlusion types. Discussion and Conclusions: the findings of this research highlight distinct three-dimensional facial morphological differences between children with Class I and II occlusions. While Class II children tended to have more convex facial profiles and less prominent lower jaws, linear measurements showed minimal variation between the two occlusion types. These results underscore the importance of three-dimensional analysis in understanding facial morphology in growing patients with different occlusal patterns.

## 1. Introduction

Facial harmony and balance arise from the interaction between both hard and soft tissue structures, but the visual appearance of the face is predominantly influenced by the arrangement and proportional distribution of the overlying soft tissue [1,2,3]. Facial soft tissue morphology plays a pivotal role in determining facial harmony and esthetics, yet its relationship with underlying skeletal and occlusal patterns remains a subject of significant interest in orthodontics and craniofacial research. The hard and soft morphologies are identified on individuality, but the maxillomandibular and occlusal relationship is only moderately manifested by the overlying soft tissue facial appearance [2]. In skeletal class II, increased overjet, and a fairly convex face, can be easily identified through the analysis of the soft tissue [2,4,5,6,7,8]. Again, malocclusion is not always associated with poor facial esthetics, and skeletal Class III malocclusion or open bite cannot be detected through cutaneous information only. Furthermore, the growth of the facial soft tissues is independent of the underlying hard tissues. Cephalometry has been extensively used and described in detail to analyze the skeletal pattern and features of malocclusions, the various categories of occlusal patterns, and their skeletal and dentoalveolar compensations [9,10].

Unfortunately, not all of these hard tissue data are directly instructive about the cutaneous relationships, and the soft tissue features associated with the various occlusal patterns must be studied separately.

Orthodontic treatment has traditionally focused on restoring the occlusion and skeletal relationship, but in recent years there has been a lot of emphasis on soft tissue improvements as well as the optimal location of them. It is extremely important to pay attention to facial structures, which should be a primary goal of treatment; balancing skeletal and dental impairments has a direct effect on their position. Soft tissue drape should be taken into consideration according to facial types considering long or short face individual patterns. Also, the compensatory nature of growth should be detected before treatment to obtain optimal therapeutic results. The assessment that correlates facial soft tissue features with the underlying skeletal structure offers a view into how deficiencies in facial appearance correspond to the existing dentoalveolar anatomy.

If the analysis of cutaneous structures could be performed with non-invasive photographic or TV styles, many individuals who would be excluded by any radiographic research could be investigated. To date, only a few two-dimensional data collected on radiographic projections have been described in the literature, and few three-dimensional data are available [8,9,10,11,12].

The use of three-dimensional data could provide a better understanding of the facial characteristics of subjects with different occlusal patterns. The three-dimensional coordinates of selected facial landmarks could be obtained by a non-invasive instrument developed for the discovery of single-label markers (ELITE) coupled with suitable algorithms [13]. The utilization of the ELITE system represents a significant advancement in non-invasive facial landmark detection, offering precise three-dimensional coordinates critical for analyzing soft tissue morphology. Previous studies evidenced promising results for a sample of 40 male and 40 female adults [14].

The instrument detects cutaneous landmarks with an infrared system, and these landmarks can be used as the endpoints for the calculation of facial angular and linear dimensions. Occlusal classes can still be classified according to Angle, using criteria that have not changed significantly in the last 90 years [14]. Angle’s orthodontic classification does not allow for the quantification of the degree of disto- or mesio-occlusion, and its description of Class I is frequently subject to confusion and inconsistency because it has too wide a range of deviations.

Katz afterward proposed a modification of the Angle occlusal classification to provide quantitative information on the occlusal connections that could allow the dentist to classify cases on a further rational basis. According to Katz’s proposal, in permanent dentition, the occlusal relationship is estimated on the most anterior premolars, while in the primary and mixed dentitions, the primary first molars or the canines can be observed [15].

The ideal occlusal relationship of the maxillary and mandibular premolar cusps, when the vestibular cusp of the most anterior maxillary premolar occludes on the distal surface of the most anterior mandibular premolar, has a deviation of zero. The deviation of the mandibular tooth cusp from this relationship is measured in millimeters, positive values indicating a more distal position of the mandibular tooth relative to its maxillary opponent and negative values indicating a more mesial position. Patients with positive deviations will have a Class II tendency, while patients with negative deviations will have a Class III tendency. As a matter of fact, in this modified classification, all subjects with any deviation from the ideal occlusal relationship should be classified as Class II or Class III.

In clinical practice, as well as for research purposes, a certain range of variation should be obtained for Class I occlusion, and the limit between the occlusal classes should be quantified. While cephalometric analyses provide valuable insights into skeletal patterns, they often lack the direct assessment of soft tissue dynamics. The utilization of three-dimensional facial landmarks in this study offers a comprehensive understanding of the relationship between soft tissue morphology and occlusal patterns, a crucial aspect not addressed solely by traditional cephalometric methods.

Clinicians, including plastic and maxillofacial surgeons, as well as orthodontists, have long been investigating the dynamics of facial development from infancy to adulthood due to the potential for disproportionate growth to result in facial disharmonies. Understanding the extent and timing of growth, its direction, and when it ends are crucial for determining treatment choices and timing.

A three-dimensional system for the landmark representation of the soft tissue facial surface has been developed and tested. This system yields genuine three-dimensional data that are unaffected by head posture or projection errors. It is also non-invasive, making it suitable for usage on young subjects [11].

The aim of this study was to update previous efforts to determine the efficacy of the system and to study a new sample of young growing patients. Also, an aim was to determine whether children with a bilateral Angle class I occlusion (modified according to Katz) differ in their soft tissue three-dimensional linear and angular facial characteristics from children of the same age with a bilateral Class II occlusion.

## 2. Materials and Methods

### 2.1. Sample and Occlusion

In total, 132 males and 137 females aged between 6 and 9 were identified from a group of 510 children attending a primary school where a cross-sectional and longitudinal growth study has been performed. The parents of the children were preliminarily informed about all the study generalities and gave their written consent to it.

The study protocol has been approved by the ethical committee for research in human subjects: UOC 420/425, Project Number 3, Year 2018, issued by IRCCS Fondazione Cà Granda, Ospedale Maggiore Policlinico, Milan, Italy. All human research procedures were followed in accordance with the ethical standards of the committee responsible for human experimentation (institutional and national) and with the Helsinki Declaration of 1975, as revised in 2013.

All patients’ parents signed an informed consent form before starting the study.

In all children, the left and right occlusal classes were measured as suggested by Katz [15].

The measurements were performed on the first premolars or the primary molars if one or the first premolars were absent in the child. In a limited number of children, the evaluation was performed on the canines, because both molars and premolars were absent according to Katz’s parameters [15].

The measurements were performed directly in the child’s mouth with a modified caliper. For the study, we used a standardized and validated modified caliper—specifically, the Mitutoyo Absolute Digimatic Caliper. The measurements were taken by two operators (N.C. and C.M.), and each measurement was repeated twice to ensure the precision and repeatability of the data. The limit between Class I and Class II, or Class I and Class III, was fixed at a 2 mm deviation; a child was therefore classified as having a Class I occlusion when he or she had the most ideal anterior premolar (or primary molar) occlusion or a deviation from this ideal occlusion between ±2 mm; a Class Il occlusion when the (distal) deviation was advanced more than 2 mm; or a Class III occlusion when the (mesial) deviation was lower than −2 mm. Only the children with a symmetric occlusal class, with same occlusal relationship on both sides of the mouth, were further considered.

The sample size was calculated according to previous studies to detect a difference of 0.1 mm RMS with a standard deviation of 0.1 mm between the two meshes; a minimum of 90 samples is required to obtain a power of 0.80 and a significance level of 0.05.

Inclusion and exclusion criteria:

- Healthy (no systemic diseases) children,
- age between 6 and 10 years,
- no craniofacial abnormalities or growth disorders,
- a symmetric occlusal class with the same occlusal relationship on both sides of the mouth,
- no orthodontic treatment in progress.

All patients meeting the eligibility criteria were included.

The sample size was calculated according to previous studies. To detect a difference of a margin error of 0.05 mm, a sample size of 90 for each group was required. This was determined with a 95% confidence level to obtain a power of 0.80. To statistically analyze the results, U–Mann–Whitney and Pearson chi-squared tests were used.

### 2.2. Collection of Three-Dimensional Facial Landmarks

For each child, the three-dimensional coordinates of 16 standardized facial landmarks were automatically collected using the ELITE system. This instrument, designed for the detection of single markers, works with specific algorithms to study cutaneous points in a non-invasive and cost-effective manner. The system can be easily utilized in orthodontic practice to provide normative data and is also suitable for longitudinal studies.

The system consists of two CCD cameras that photograph the child, real-time hardware for the recognition of markers, and software for the three-dimensional reconstruction of the x, y, end z coordinates of the landmarks relative to the reference system [11,13].

The system and the used protocol have been described in detail previously [13].

**Figure 1:**
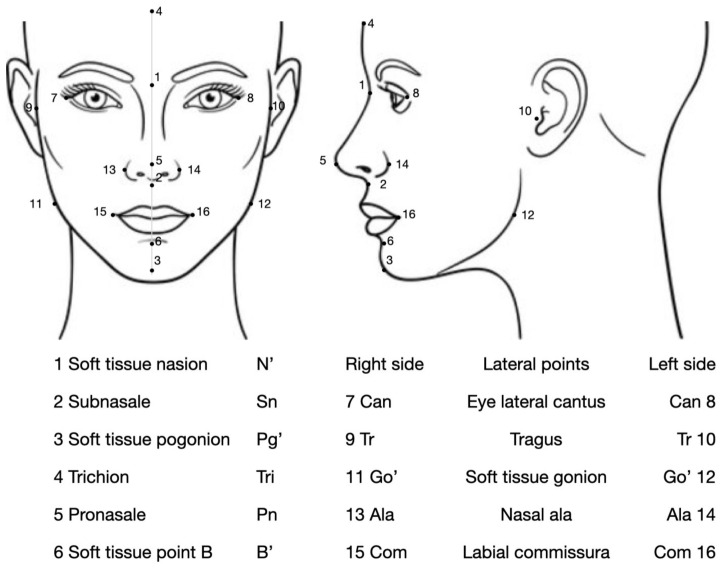
Identification of the considered landmarks.

Each patient was asked to sit in a chair with their head in a natural position. The 16 landmarks were identified through careful inspection and/or palpation and then marked on the face of each child using black eyeliner (Figure 1).

- Midsagittal plane points: trichion (i); soft tissue nasion (the innermost point between the forehead and nose) (i/p); pronasale (nasal apex) (i); subnasale (i); soft tissue B′ point (i/p); and soft tissue pogonion (the most prominent point on the chin) (i).
- Left and right side points: eye lateral canthus (on the frontozygomatic suture) (i/p); nasal ala (i); labial commissure (i); tragus (i); and soft tissue gonion (i/p).

On the center of each point, a 2 mm spheric reflective marker was later fixed with an adhesive plaster. The same operator identified and located all the points. Each child was seated in centric occlusion (natural occlusal contact without clenching force) in an upright position in front of two cameras positioned to obtain a stereometric view of the child’s face. The CCD cameras illuminated the reflective markers with an infrared stroboscope, automatically recognizing and recording the centers of gravity of these points. The ELITE system then provided the spatial x, y, and z coordinates of the 16 points.

### 2.3. Facial Morphometry

The x, y, and z coordinates of the 16 points collected on each child were used to calculate many angles and linear measurements, as well as some distance ratios (Table 1). All the measurements were obtained in the three-dimensional space, i.e., the positions of the points relative to all three planes (frontal, lateral, and vertical) were considered at the same time (no projections). The children were subdivided into three age classes. For each age class (Table 2), girls and boys were pooled, and mean values were calculated separately for children with a bilateral Angle Class I occlusion (modified according to Katz”) and for children with a bilateral Class II occlusion. Mean angular values were calculated with dedicated statistics for circular variables, as detailed by Batschelet, by using the rectangular factors of each angle [15]. The same system allowed for the calculation of relevant dispersion measures (the standard angular deviations). Standard angular deviation has the some meaning as standard deviation has in linear statistics. Comparisons between the mean direct values and ratios computed for the Class I and Class II groups within each age class were performed with the Student’s t test, while angular values were compared with Watson Williams’ test.

The significance level was set at 5 (*p* < −0.05). All of the calculations were performed on a Z-Stalion 466 Xn (Zenith Data System) with original computer programs.

### 2.4. Data Collection Error

To assess the reproducibility of the morphometric data collection procedure (ELITE system and mathematical reconstruction of point coordinates), the facial landmarks of five children (two males and three females) were identified and collected [1] twice with the same ELITE calibration and with two different ELITE calibrations. The combined error of the ELITE system and computer programs (where differences could arise from the approximation algorithms) was estimated to be about 0.1 mm for all three point coordinates (x, y, and z).

## 3. Results

Table 1 reports the number of children with bilateral Angle Class I or Class II occlusions (modified according to Katz) together with the relevant percentages of the total number of children of that age class. In all three age classes, about 50% to 65% of the children had a symmetric Class I occlusion, i.e., their maxillary and mandibular premolars or primary molars had an ideal or almost ideal relationship. About 15% to 20% had a Class II occlusal relationship on both sides, while 19% to 30% had asymmetric occlusal relationships. Only one child had a bilateral Class III occlusion, even if the facial morphometric characteristics were Class I or Class II.

The three-dimensional soft tissue facial characteristics of these children were therefore analyzed using the equals of 16 selected facial landmarks. The mean values were computed within the age and occlusal class. Sex was not considered as a factor because, in a former investigation performed on the same children, no statistically significant differences were identified between males and females of a similar age as far as the measurements listed in Figure 1 were concerned [15,16,17].

Table 2 reports the statistically significant differences between Class I and Class II children; most of the differences involved three-dimensional angular measurements. At all the considered ages, Class II children had a more convex face in the sagittal plane, with lower soft tissue facial convexity angles, measured at both the subnasale and pronasale, and a larger maxillary prominence angle. All the measurements used are presented in Table 3.

In two of the three age classes, the mandibular plane angle Go′-Pg′-Go′ was larger in Class II than in Class I children. Also, in the horizontal plane, a lower mandibular prominence was correlated with a distal occlusion. Among the children aged 7 to 8 years old, the left gonial angle was significantly larger in Class I than in Class II children; in the same individualities, an analogous difference influenced the mandibular ramus height (mean left- and right-side values). No other differences were identified for the linear measurements. Only one linear distance rate was different between the two groups in the two age classes, but the difference was not consistent; the lower facial height rate was larger in Class I children aged 6 to 7 years, but smaller in Class I children aged 7 to 8 years, than in Class II children of comparable ages.

## 4. Discussion

Over the years, researchers have utilized various methods, including cephalometric radiographs, photographs, and 3D images/facial scans, to evaluate soft tissue for orthodontic diagnosis.

While CBCT images and 3D face scans offer more accurate results and facilitate measurements, their routine use in orthodontic patients is limited due to the high radiation dose associated with CBCT images and the cost of 3D face scans. The procedure proposed in this study can aid in performing anthropometric measurements directly on the face of the patient to assess soft tissue morphology in an easy and safe way. In fact, the ELITE system can be accurate enough for soft tissue diagnosis and patient communication, and it could also be accurate in predicting soft tissue changes after therapy. This approach offers not only a clear and intuitive representation of soft tissue drape but is also a valuable alternative to direct anthropometry, minimizing identification and repetitive errors.

Furthermore, it decreases selection bias and unreliable results by avoiding the inaccurate evaluation of individual soft tissue.

Nevertheless, the findings of this study enable clinicians to better visualize and gain more insights into the facial soft tissue to make decisions when planning orthodontic therapy, particularly for patients with esthetic requirements. Clinicians can thereby strive to increase the validity of orthodontic measurements and patient satisfaction after the treatment.

In this study, three-dimensional cutaneous facial morphometry was performed in a large group of healthy children. A non-invasive system was applied, and it allowed for the automatic identification of linear and angular dimensions and the analysis of the relationships among the different facial soft tissue structures. In all children, the Angle occlusal class, modified according to Katz, was measured directly in the mouth. Approximately one-third of the children in the different age groups had a bilateral Class I occlusion; accordingly, in a former study, Angle Class I occlusion was found in 60% of the dental casts of healthy, youthful, grown-up young adults with a complete permanent dentition [14].

The distribution of occlusal classes was similar in another study, with no differences for gender, and no differences were set up between the linear and angular facial data collected from males and females of the same age class and with the same occlusal relationship [17].

Combined male and female data were therefore used in the present study.

Also, Athanasiou et al. found no significant differences in the cephalometric measurements taken in children of a similar age, and the sexual dimorphism for soft tissue growth identified by Nanda et al. and Farkas and Posnick did not include children between 6 and 9 years in most of the present measurements [18,19,20].

The results of the present study demonstrated that altered occlusal and skeletal relationships impact the soft tissue appearance, and the angular facial characteristics are more affected than the linear dimensions. The cutaneous tissue partially masks the underlying skeletal imbalances: the strong correlations between the Angle Class of malocclusion and the skeletal vertical facial dimensions found by Siriwat and Jarabak are not reflected by the soft tissues, but the soft tissue profile of Class II children is more convex than that of Class I children (smaller soft facial convexity angles and larger maxillary prominence angles (see Table 3)) [10,11,12,13,14,15,16,17,18,19,20,21,22,23].

The soft tissue maxillary prominence angle (B′-N′-Sn) measured in our investigation can be considered to be the soft tissue analog of the skeletal point A-nasion-point B angle, even if only a moderate correlation between the two angles exists [2].

This angle (B′-N′-Sn) was found to be consistently larger in Class II children than in Class I children at any age. Class II children were also found to have a larger mandibular plane angle (Go′1-Pg′-Go′) than Class I children, showing a less prominent mandible also in the vertical plane.

The present three-dimensional results agree with the findings of the study in which the soft tissue characteristics of Class I and Class Il children were quantified [8]. The observed discrepancies between soft tissue and skeletal relationships emphasize the complex interplay between underlying skeletal structures and overlying soft tissues in determining facial esthetics.

In their radiographic two-dimensional investigation, Genecov et al. showed that children with a skeletal Class II occlusion had a more retrusive soft tissue chin and a more anterior position of the nasal dorsum than did children of a comparable age with a Class I occlusion [24].

Another study [25] provided an overview of the differences with standard facial scanning and facial features retrieved from the CBCT, providing interesting insights on the topic. Images with scanned soft tissues, crucial for orthodontic diagnosis and patient safety, can be retrieved from both facial scans and CBCT with similar cephalometric results, as has been described. The results indicated a mean root median square (RMS) difference of 1.8 mm between the CBCT-derived and facial scan-based soft tissue representations. The angular measurements exhibited minor differences, while the linear measurements showed a lower correlation.

Recently, several authors have highlighted the changes in facial morphology following orthodontic therapy, particularly in patients with a class II malocclusion, which is often accompanied by skeletal mandibular retrusion, or in patients with alterations in the anterior vertical dimension [25,26,27,28,29,30,31]. Functional therapy promotes mandibular reposition through the forward posture of the mandible as well as backward or anterotation, with the condyles more in a downward and forward position within the glenoid fossa [31,32,33,34]. The primary changes are an efficient mandibular development with a decreased maxillary forward development and the reposition of upper and lower incisors [9,35]. Although the skeletal and dental effects have been well documented, there are a lack of data on soft tissue changes [35,36,37,38,39]. The few results available suggest that the soft tissue facial structures should be better studied as they are affected by orthodontic appliances. Some studies have indicated the soft tissue response in the short term with orthodontic appliances [40,41,42,43,44]. To be able to analyze them in a non-invasive and simple manner before treatment, the orthodontist should be given suggestions on how to treat the patient to improve them.

Facial harmony in orthodontic treatment is a crucial element for treatment success, and it is essential in order to plan the most effective treatment. Thus, the accurate prediction of how soft tissue could change after treatment could help greatly in determining treatment sequences. Having instruments to analyze them before treatment can aid in selecting various treatment strategies to formulate an appropriate diagnosis in order to plan a clear goal. In orthodontic diagnosis and treatment planning, it is important to assess each patient’s malocclusion, considering all structures of the stomatognathic apparatus, including soft tissue morphology. While cone-beam computed tomography (CBCT) has emerged as a valuable tool for acquiring three-dimensional evaluations of maxillofacial morphological features, its routine use is limited due to concerns regarding radiation exposure [34,35,36,37,38,39,40,41,42,43,44,45,46]. Therefore, the lateral cephalogram is more commonly used for skeletal and dental assessment, indispensable in aiding in the understanding of the morphology of malocclusion. All these methods are precise enough to analyze skeletal and dental components but are not precise enough to evaluate soft tissue morphology.

The method applied in the present investigation allowed for a precise quantification of facial soft tissue that was estimated in its three-dimensional aspect and not on separate two-dimensional projections. Indeed, this system allows for the measurement of actual distances and angles, overcoming all the problems of the projection and superimposition of structures that afflict the most extensively used radiographic cephalometry. Because it is non-invasive, it can be easily employed in longitudinal studies following the effects of growth and treatment on the soft tissue of patients with selected malocclusions. We think a non-invasive method could represent an opportunity in the future, perhaps when a traditional analysis is not suitable. Future research may benefit from exploring the longitudinal changes in facial soft tissue morphology following orthodontic interventions, evidencing the dynamic nature of facial esthetics and stability throughout growth influenced by the treatment [46,47,48,49,50]. This study has some limitations that should be addressed. The soft tissue traits of the face in fact can be affected by some confounders, ranging from genetics and illnesses to environmental factors. Syndromes, for example, could alter the facial features or morphology. Furthermore, the limited presence of class III patients should be addressed, even if the prevalence in children is low. Also, the inter-rater variability was not measured for this study. In addition, a long-term follow-up investigation of the craniofacial morphology of these groups should be conducted in future studies for a more comprehensive study.

## 5. Conclusions

In this study, the three-dimensional analysis of facial soft tissue morphology in healthy children demonstrated that changes in occlusal and skeletal relationships may affect facial appearance, with angular facial characteristics being more affected than linear dimensions. We believe that explaining this aspect to the parents of young patients is crucial for conveying the importance of orthodontic care. Children with a Class II occlusion exhibited a more convex facial profile compared to those with a Class I occlusion, showing distinct differences in soft tissue angles. This non-invasive approach enables the accurate assessment of facial soft tissue longitudinally, facilitating the study of growth and treatment effects in patients with malocclusions. This technology can, and hopefully will, be implemented with the use of 3D software and new facial scanners. Further studies are needed to standardize its usage protocol.

## Figures and Tables

**Table 1:** Class I and II morphometric profile among the sample analyzed.

Age (y)			
	6.7	7.8	8.9
Boys	45	44	43
Girls	33	55	49
Total group	78	99	92
Bilateral Class I	53	49	48
Percent of total	41.34	48.51	44.16
Bilateral Class II	18	25	19
Percent of total	14.04	24.75	17.48

**Table 2:** Statistically significant differences between Class I and Class II children divided by their ages.

	Class I		Class II		Comparison	
	Mean	SD	Mean	SD	Ft/t	*p*
6 to 7 years old						
Angles (°)						
1 N′-Sn-Pg′	160.09	5.2	156.03	6.55	4.55	<0.05
2 N′-Pn-Pg′	134	4	131.08	6.53	4.22	<0.05
3 B′-N’-Sn	8.78	2.11	10.87	2.55	6.55	<0.025
8 Go′-Pg’-Go′	75.67	3.94	79.04	5.94	4.8	<0.05
Linear distance ratios %						
4 Tr-Go′/Sn-Pg′	118	9.4	110.57	13.55	2.02	<0.05
7 to 8 years old						
Angles (°)						
1 N′-Sn-Pg′	160.41	4.07	157.34	4.27	8.93	<0.05
2 N′-Pn-Pg′	133.85	3.67	130.98	3.2	7.28	<0.01
3 B′-N′-Sn	8.7	1.71	10.38	1.93	10.55	<0.05
10 Tr′-Pg’-Go′	127.3	5.66	129.97	4.07	5.85	<0.025
Linear distance (mm)						
15 Mandibular ramus height	48.9	3.69	51.84	3.23	2.88	<0.01
Linear distance ratios %						
4 Tr-Go′/Sn-Pg′	115.81	11.29	123.8	12.85	2.34	<0.025
8 to 9 years old						
Angles (°)						
1 N′-Sn-Pg′	161.04	4.13	156.7	2.7	12.78	<0.005
2 N′-Pn-Pg′	133.93	3.94	130.94	5.49	4.72	<0.05
3 B′-N′-Sn	8.29	1.79	10.7	1.41	19.25	<0.005
8 Go′-Pg’-Go′	75.89	4.11	79.23	5.11	5.9	<0.025

**Table 3:** The linear and angular measurements used.

Linear Measurements	Angular Measurements
Tri-N′ Tri-n′ N′-Sn N′-Sn Sn-Pg′ Sn-Pg′ N′-(Tr-Tr) Sn-(Tr-Tr) Pg′-(Tr-Tr) Can-Can Tr-Tr GO′r-GO′ Com-Com Pg′-(GO′r-GO′~) Tr-Go′	N′-Sn-Pg′ N′-Pn-Pg′ B′-N′-Sn′ Tq-N′-Trr Tq-Pn -Tr Tr1-Pg′-Trr Can-N′-Cant Go′a-Pg′-Go′r Trr-Go′r -Pg′ TrI-Go′1-Pg′

## Data Availability

The data can be requested from the corresponding author upon reasonable request.
